# Author Response: K320E-Twinkle^skm^ Mice Are Genetically Heterogeneous for Secondary mtDNA Deletions Impairing Comparison With Controls

**DOI:** 10.1167/iovs.62.1.13

**Published:** 2021-01-11

**Authors:** Sammy Kimoloi, David Pla-Martín, Rafael R. Oexner, Olivier R. Baris, Rudolf J. Wiesner

**Affiliations:** 1 Center for Physiology and Pathophysiology, Institute of Vegetative Physiology, University of Köln, Köln, Germany; 2 Department of Medical Laboratory Sciences, Masinde Muliro University of Science and Technology, Kakamega, Kenya; 3 Cologne Excellence Cluster on Cellular Stress Responses in Aging-associated Diseases (CECAD), University of Köln, Köln, Germany; 4 Equipe MitoLab, UMR CNRS 6015, INSERM U1083, Institut MitoVasc, Université d'Angers, Angers, France; 5 Center for Molecular Medicine Cologne, University of Köln, Köln, Germany. E-mail: kimoloi@mmust.ac.ke.

**Keywords:** mtDNA deletions, extraocular muscle, type IIB fibers

We thank J. Finsterer for his comments on our recently published paper,^1^ in which we used myosin light chain 1f (Mlc1f) driven Cre-recombinase to conditionally express only in skeletal muscles the dominant-negative K320E-Twinkle mutant helicase, the human analogue causing SANDO syndrome.^2^ Like in patients with progressive external ophthalmoplegia, this and other mutant Twinkle helicases generate mutations in a stochastic manner with unpredictable, arbitrary breakpoints, thus leading to the accumulation of multiple deletions^3^ but also gene duplications (Fig. 1B, smear of long range PCR products),^1^ confirmed recently in our mice by a new approach (Basu et al., 2020). Thus, those animals do not accumulate single deletions that could account for phenotypic heterogeneity, as suggested by J. Finsterer. We did not investigate the heteroplasmy rate of mitochondrial DNA (mtDNA) deletions in this study, but expression of this dominant negative form of TWINKLE in mouse hearts leads to a proportion of 85% of deleted mtDNA molecules in COX deficient (COX^−^) cardiomyocytes at 18 months of age (Baris et al., 2015). The deletions 1, 3, 13, and 17 that we quantified as examples are among the most common deletions reported in aging mice^4^ and even under regular PCR conditions, primers yielded multiple products (Fig. 1D).^1^ Therefore, each of the fibers has an equal chance to acquire and accumulate multiple mtDNA mutations, irrespective of its type, and no specific fiber type is expected to be disproportionately affected. However, our data clearly show that the number of type IIB fibers exhibiting COX deficiency is disproportionately higher in comparison to other fiber types.^1^ Accordingly, this points to fiber type IIB specific factors or physiology that selectively increases their vulnerability to mtDNA alterations.

The higher prevalence of COX deficient fibers in the global layers of the extraocular muscles (EOMs) is certainly due to the higher proportion of the vulnerable type IIB fibers compared to the orbital layer. This is further supported by the higher proportion of COX deficient fibers in the fiber type IIB rich retro bulbar (RB) muscles.^1^ If differential movement is a factor, as suggested by J. Finsterer, then differences between inter-recti and global layer would be apparent, but we did not detect such differences.

We also disagree with his notion that increasing amounts of mtDNA alterations rather than COX deficiency drives the reduction of type-IIb fibers and alteration of fiber type composition in EOM. We believe that as the proportion of mtDNA alterations increases, the severity of the associated respiratory chain defects increases too, ultimately causing death of the affected fibers.

Finally, we would like to re-emphasize that our model expresses the mutant K320E-Twinkle specifically in mature muscle fibers, and consequently, we did not investigate phenotypic changes in other, nonexpressing organs. In addition, chronic progressive external ophthalmoplegia (CPEO) is usually referred to as a mitochondrial myopathy disease that is mainly accompanied by clinical symptoms of skeletal muscle weakness (most prominently ophthalmoplegia but also proximal limb weakness). Therefore, even though it is not comprehensively covering the broad and continuous phenotypic spectrum of other human mtDNA deletion syndromes, we strongly believe that the K320E-Twinkle^skm^ mouse is a valuable model for CPEO.
